# How do Chinese people evaluate “Tang-Ping” (lying flat) and effort-making: The moderation effect of return expectation

**DOI:** 10.3389/fpsyg.2022.871439

**Published:** 2022-11-16

**Authors:** Han-Yu Hsu

**Affiliations:** School of Social Development, East China University of Political Science and Law, Shanghai, China

**Keywords:** effort-making, improvement belief of effort, obligation belief of effort, return expectation, Tang-Ping

## Abstract

“Tang-Ping” (TP), referring to “lying flat” literally, has been a buzzword in China web media since 2021. As the opponent of effort-making (EM) behaviors which have both instrumental and purpose values in Confucian culture, TP has a negative moral implication in China and has been criticized by the state-owned media. Meanwhile, the meaning of TP also contains a negative form of resistance toward social and organizational inequality, which may be acceptable under unfair circumstances. This study employed the imagined-scenario method to investigate the public’s moral evaluations of TP and EM behaviors under conditions of different return expectations. An online questionnaire with 2 (TP vs. EM) by 2 (low vs. high return expectation) between-participants designed scenarios were employed, along with the measurements of obligation belief of effort (OBE) and improvement belief of effort (IBE) scales (*N* = 210). The results found that (1) TP behaviors were evaluated as morally wrong in general, while EM behaviors were morally right; (2) the return expectation of the scenario moderated the behavior type’s effect on moral evaluation, that EM behaviors were evaluated positively regardless of return expectation, while TP behaviors became acceptable with a neutral score under the low return expectation; (3) both OBE and IBE correlated positively with evaluations of EM while negatively with evaluations of TP. The theoretical and practical implications were discussed.

## Introduction

*Tang-Ping* (躺平, TP for short), meaning “lying flat” literally, has become a buzzword in Chinese web media, which refers to a simple lifestyle without effort making. In April 2021, a post titled “TP Is Justice” at *Baidu Tieba* (a Chinese internet forum) described the poster’s lifestyle, which he called TP. In the now-deleted post, the poster said he had had no full-time work for 2 years, only done part-time with 200 CNY income monthly (about 32 USD). He tried to keep an elementary life without any intention of marriage, parenthood, or apartment purchase, which were typical stressors in contemporary China (Wikipedia Contributors, 2022). The poster also cited ancient Greek cynicism philosophy to interpret his behaviors as “I could sleep in my jar and sunbath as Diogenes,” which reflected the term of modern Kynicism that “*rejection of the official culture by means of irony and sarcasm*” (Žižek, 2009; Ling and Li, 2022).

After this post, the meaning of TP has been extended to a negatively resistant behavior toward social competition. Most official media in China criticized the saying and phenomenon of TP, such as an editorial, “*‘Lying flat’ shameful, where is the sense of justice*” (‘躺平’可耻, 哪来的正义感), by state media Xinhua (Wang, 2021). Besides criticizations, a few Chinese media and scholarships also mentioned the social structural background behind TP: High pressure in apartment-purchase and child-parenting, over-competitions in education and the workplace, and low return rate after effort making (Xiang, 2021; Ling and Li, 2022). Under this relatively unfair circumstance, some Chinese people started to give up part of their ambition and desire and chose TP instead of struggling and making efforts. All the published media and scholarships, no matter with a criticized or sympathetic stand, indicated that TP is opposite to effort-making (EM) behavior and the doctrine of self-exertion as a Confucian cultural value (Fwu et al., 2017).

Existing literature has discussed the relationship between cultural values and EM behaviors in academic learning (e.g., Li, 2012; Fwu et al., 2017). From the perspective of Chinese cultural psychology, EM has both instrumental value for individuals’ self-improvement and purpose value as individuals’ obligation to fulfill. For the latter, Confucian culture regard EM as a way to self-exertion (*Jin-Ji*, 尽己), and lack of EM is viewed as morally wrong (Fwu et al., 2014). Li (2012) also argued that learning with effort is “virtue-oriented” with moral significance in Eastern cultures while “mind-oriented” in the West. Hwang (2012) compared Confucian and western ethics and argued that academic EM is a kind of *unconditional positive duty* that asks individuals to behave actively and regarding not doing so as a sin, distinguished from Kant’s *perfect duty of omission* or *imperfect duty of commission*.

To conceptualize and quantitatively measure the internalized cultural value of EM in academic learning, Chen et al. (2016) proposed the *Beliefs about Effort* theory, that Chinese people hold two kinds of effort beliefs at the same time: *Obligation-oriented belief of effort* (OBE) that it is someone’s obligation to make an effort on learning and *improvement-oriented belief of effort* (IBE) that effort can help to conquer one’s ability limitation and to improve one’s academic performance. In empirical research, OBE and IBE showed different functions. When students experienced a failure in which they did not get the expected return of achievement, OBE positively predicted the striving behaviors after failure. In contrast, IBE did not have this effect (Chen et al., 2019). As an internalized cultural value, OBE emphasizes continuous EM behavior as a virtue even with failure (low return), while IBE regards EM as a method for ability improvements and achievements. Although the *Beliefs about Effort* theory was developed in the academic field, cultural values may still influence people’s evaluation of EM and TP behaviors in the non-academic workplace, which will be explored in the current research.

In the organizational and working context, EM behaviors are also encouraged by cultural values. However, the representation of the return of EM is different from that in the academic and learning context. In the learning context, students’ EM behaviors are targeted at their own improvement and achievement in the academic field. However, in the workplace, although workers also need to improve their working skills, such improvements are also instruments for their working tasks. Along with workers’ self-improvement of personal skills and careers, workers’ EM behaviors are also their social exchange with the administrative authorities, paying labor in exchange for a fair salary (Cooper and Scandura, 2015; Hussain and Shahzad, 2022). Compared to the academic field, whether there would be a fair return may influence individuals’ evaluation of EM behaviors. Past research demonstrated multiple forms of organizational justice, including distributive and procedural justice (Tyler, 1994). In this article, we focus on distributive justice about whether the EM behavior could get a fair return or not.

Considering this distinction, people’s evaluation process of EM and TP behaviors may also differ in academic and working situations. As mentioned before, EM in the academic field is an *unconditional positive duty* (Hwang, 2012), while not EM is a sin (Fwu et al., 2014). But what about TP (not EM) in the workplace? If a worker chooses to TP when the organization does not offer a fair return, do the observers in a Confucian culture still evaluate TP as morally wrong? We infer that, compared to the *unconditional positive duty* in the academic context, EM would be a kind of *conditional positive duty* (i.e., Kant’s imperfect duty, refer to Hwang, 2012, for a review of duty theories), and that EM is a virtue but not EM is just lack of virtue while not a sin. The condition of EM as a positive duty in the workplace would be organizational fairness, which would be operationalized as the return expectation in our empirical research.

Meanwhile, EM in the workplace is still a way for self-exertion; thus, the internalized cultural values may still function in the moral evaluation process toward EM and TP behaviors in the workplace. According to Chen et al. (2016), OBE emphasizes the purpose value of EM itself, while IBE emphasizes the instrumental value of achievements. Therefore, OBE and IBE would both correlate positively with evaluations of EM and negatively with that of TP. At the same time, the correlations of OBE-EM/TP should be more stable regardless of return expectation, while IBE-EM/TP should be more dependent on the fairness of circumstance.

In our previous qualitative research (Hsu, 2022), Chinese people’s understanding of TP had a precondition of high competition with low return expectations within social and organizational contexts. Although most participants regarded TP with negative attitudes, some people stated a positive form of TP as a circuitous resistance and sympathized with it. The English version of TP’s phenomenography can be found in Supplementary material.

Meanwhile, how ordinary people evaluate TP, its relationship with cultural values of efforts, and the condition of return expectation are still unclear. Based on previous research on EM and the current social issue of TP, the present article aims to extend the applied range of Chinese cultural theory about EM to a more general social context. By the quantitative method of imagined scenarios, empirical research was designed to investigate the public’s moral evaluation of TP and EM behaviors under different return expectation conditions. Participants’ effort beliefs (OBE and IBE) were also measured. Three hypotheses were proposed:

H1: In general, TP would be evaluated worse than EM.

H2: EM would be evaluated positively under both high and low return expectation conditions for the existence of virtue value under low return expectation.

H3: TP would be evaluated less negatively under high return expectation conditions than low for the absence of instrumental value.

H4: In general, evaluations of EM are positively correlated with OBE and IBE, while evaluations of TP are negatively correlated with OBE and IBE.

H5: Correlations of OBE-TP/EM are more stable than IBE-TP/EM, that OBE correlated with evaluation of TP/EM regardless of return expectation. In contrast, IBE would correlate with TP/EM higher under high return expectation conditions than low return expectation conditions.

## Methods

### Participants and procedure

An online questionnaire survey was conducted at www.credamo.com with its national representative sample pool in China. After excluding 76 invalid responses which failed in the manipulation check items, 210 Chinese people participated in this study (Age: *M* = 29.81, *SD* = 6.38; Gender: 53.8% women, 46.2% men; Education: 2.9% high-school and below, 85.7% bachelor, 11.4% graduate).

After reading the informed consent, participants were invited to complete an anonymous questionnaire that lasted 10–15 min. The effective response providers could get five CNY rewards, and the staff of www.credamo.com executed the payment process with desensitization from research data. All questions were presented in Chinese.

### Instrument

#### Scenario questions

Four scenarios were constructed to investigate the effect of behavior types (TP vs. EM) and return expectations (low vs. high), followed by two evaluation questions. The original Chinese versions of the scenarios can be found in the Supplementary material, and the English translations are as follows:

Version 1 (EM with low return expectation): *Zhang graduated from university recently and entered an internet company. The company provided high amounts of annual performance bonuses, but Zhang heard that few colleagues had gotten it. After entering the company, Zhang not only accomplished his responsibilities but also participated in additional project team jobs, did voluntary overtime work, whose performance was among the top.*

Version 2 (EM with high return expectation): *Zhang graduated from university recently and entered an internet company. The company provided high amounts of annual performance bonuses, and Zhang heard that many colleagues had gotten it. After entering the company, Zhang not only accomplished his responsibilities but also participated in additional project team jobs, did voluntary overtime work, whose performance was among the top.*

Version 3 (TP with low return expectation): *Zhang graduated from university recently and entered an internet company. The company provided high amounts of annual performance bonuses, but Zhang heard that few colleagues had gotten it. After entering the company, Zhang only accomplished his responsibilities, seldom participated in additional project team jobs, did no unnecessary overtime work, whose performance was just so-so.*

Version 4 (TP with high return expectation): *Zhang graduated from university recently and entered an internet company. The company provided high amounts of annual performance bonuses, and Zhang heard that many colleagues had gotten it. After entering the company, Zhang only accomplished his responsibilities, seldom participated in additional project team jobs, did no unnecessary overtime work, whose performance was just so-so.*

The between-participant design was employed. Each participant was randomly assigned to one version. After reading the scenario, participants were asked to answer two questions: Q1 of the evaluation of others’ behavior: “*do you agree with Zhang’s way of working?*” Q2 of the behavior tendency of oneself: “*if you were Zhang, would you behave as his way?*” Both were scored by Likert 5-point scales ranging from −2 (strongly disagree/absolutely will not) to 2 (strongly agree/absolutely will).

#### Beliefs of effort scales

To measure participants’ cultural beliefs about efforts in non-academic fields, eight items (including 4 of OBE and 4 of IBE) in the Chinese version of the Beliefs of Effort Scale (Wang and Chen, 2020) were selected and modified. The wording in the original scales of “student” and “learning” was replaced by “people/everyone” and “working,” e.g., “*hard-working is everyone’s duty*” (OBE_1) and “*everyone could overcome his/her difficulties in work if working hard*” (IBE_1). The revised and corresponding original items can be found in the Supplementary material. All the items were scored by Likert 5-point scales ranging from −2 (strongly disagree) to 2 (strongly agree). The revised OBE and IBE scales show high internal consistency (α_*OBE*_ = 0.80, α_*IBE*_ = 0.84).

## Results

### Descriptive statistics and correlations

The descriptive results of the four conditions are presented in Table 1, with correlations with OBE and IBE scales. The evaluations of EM are all positive, while evaluations of TP are neutral and negative, indicating Chinese people’s general attitude toward these two behaviors. The scores of OBE and IBE are both significantly higher above 0 (*M*_*OBE*_ = 1.23, *SD*_*OBE*_ = 0.64, *p* < 0.01; *M*_*IBE*_ = 1.00, *SD*_*IBE*_ = 0.81, *p* < 0.01) and correlated moderately with each other (*r* = 0.63, *p* < 0.01), indicating modern Chinese people’s general endorsements of traditional values of efforts.

**TABLE 1:** Descriptive and correlational results.

Target behavior	Return expectation	Q1 *Mean* (*SD*)	Corr Q1-OBE	Corr Q1-IBE	Q2 *Mean* (*SD*)	Corr Q2-OBE	Corr Q2-IBE
Tang-Ping	Low (*N* = 49)	−0.06 (1.31)	−0.29*	−0.29*	−0.10 (1.45)	−0.38**	–0.28
	High (*N* = 54)	−0.81 (1.08)	−0.64**	−0.61**	−1.07 (1.16)	−0.64**	−0.56**
Effort-making	Low (*N* = 52)	1.02 (0.85)	0.40**	0.48**	0.87 (1.10)	0.35*	0.60**
	High (*N* = 55)	1.20 (0.59)	0.20	0.15	1.16 (0.76)	0.40**	0.22

Q1: “do you agree with Zhang’s way of working?” Q2: “if you were Zhang, would you behave as his way?” *: 0.01 < *p* < 0.05. **: *p* < 0.01.

The evaluations of EM have positive correlations with beliefs of efforts, while TP is negative in general, although some are insignificant. Hypothesis 4 is partially supported. Since the sample sizes were relatively small within each condition, the OBE/IBE’s differences in correlating with moral evaluations under different conditions are not tested. However, according to the numerical values of *r*s, the patterns of OBE-TP/EM and IBE-TP/EM correlations are not differentiated. Hypothesis 5 is not supported.

## ANOVA

**FIGURE 1:**
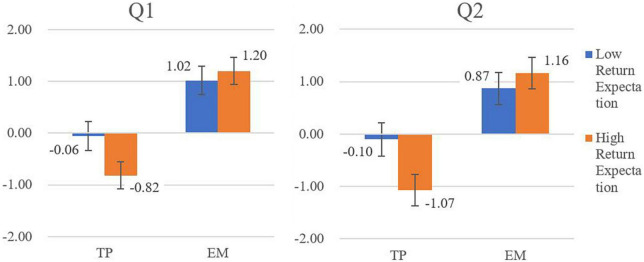
Three-way interaction between behaviors, return expectations, and questions. Error bars indicate 95% CI. TP, Tang-Ping; EM, effort-making. Q1: “do you agree with Zhang’s way of working?” Q2: “if you were Zhang, would you behave as his way?”

A 2 (Behaviors: TP vs. EM) by 2 (Return Expectations: low vs. high) by 2 (Questions: Q1 vs. Q2) mix-design ANOVA on the evaluation scores of behaviors was executed (Table 2 and Figure 1).

**TABLE 2:** ANOVA result.

Factors	*df*	*F*	*p*	partial η ^2^
**Between participants factors**				
(TP-EM)	**1**	**133.22**	**< 0.01**	**0.39**
Return expectation	**1**	**5.22**	**0.02**	**0.03**
(TP-EM) × Return expectation	**1**	**16.31**	**< 0.01**	**0.07**
**Within participants factors**				
(Q1-Q2)	**1**	**5.03**	**0.03**	**0.02**
(Q1-Q2) × (TP-EM)	1	0.25	0.62	0.00
(Q1-Q2) × Return expectation	1	0.22	0.65	0.00
(Q1-Q2) × (TP-EM) × Return expectation	1	2.36	0.13	0.01

Bold characters indicate significant effects at the 0.05 level. TP, Tang-Ping; EM, effort-making. Q1: “do you agree with Zhang’s way of working?” Q2: “if you were Zhang, would you behave as his way?”

The behavior type shows significant main effect with largest effect size [*F*(1, 206) = 133.22, *p* < 0.01, partial η^2^ = 0.39], and EM behaviors are evaluated better than TP. Hypothesis 1 is supported.

The return expectation shows significant main effect [*F*(1, 206) = 5.22, *p* = 0.02, partial η^2^ = 0.03], and significant interaction with behavior type [*F*(1, 206) = 16.31, *p* < 0.01, partial η^2^ = 0.07]. As in Figure 1, return expectation does not influence the evaluation of EM [simple main effect: *F*(1, 105) = 2.80, *p* = 0.10, partial η^2^ = 0.03], but does influence the evaluation of TP [simple main effect: *F*(1, 101) = 13.46, *p* < 0.01, partial η^2^ = 0.12], and that TP is acceptable under low return expectation (*M* = −0.82, *SD* = 0.17) while morally wrong under high return expectation (*M* = −0.94, *SD* = 0.16). Hypotheses 2 and 3 are supported.

Besides, the question type also shows a significant but small main effect [*F*(1, 206) = 5.03, *p* = 0.03, partial η^2^ = 0.02], and the scores on Q1 (agreement of Zhang’s behavior, *M* = 0.34) are slightly higher than Q2 (behavior tendency as Zhang, *M* = 0.21), while the interaction terms between question type and other IV are all insignificant. Since there is little theoretical meaning in combining different behavior types (i.e., the main effect of question type including both TM and EM behaviors), the implication of this result would not be discussed.

## Discussion

The present study examined the Chinese public’s moral evaluation of the behaviors of TP and EM under different return expectations. As a violation of the traditional value of efforts in China, TP got negative moral evaluations in general by public observers, in contrast with the EM behaviors. Meanwhile, when EM behaviors could get positive evaluation regardless of the return expectation, the TP behaviors were acceptable and understandable under low return expectations presented by neutral scores.

These results reveal the similarities and distinctions between the media and scholarship comments and ordinary Chinese people’s understanding of the TP phenomenon. Just as the Chinese state media’s criticism, ordinary people also showed disapproval attitude toward the passive working attitudes of TP. However, such disapproval attitudes were conditioned in an organizational environment with procedural and distributive justice (Tyler, 1994), which was seldom mentioned in Chinese media’s commentaries (some international media discussed this background, such as BBC, see Allen, 2021). In the current research, we found the moderation role of distributive justice (manipulated as return expectation) in the moral evaluation process. When the environment is unfair that individuals’ efforts could hardly get returns of equal value, as the manipulation of low return expectations in the quasi-experiment, the behaviors of TP may become a reasonable way to cope with social pressure, which is accepted by the Chinese public. Hard-working ethics are worthy of being encouraged, but social and organizational unfairness also needs to be criticized and reformed. Furthermore, the cultural value of efforts may play a role in concealing social contradictions, which need to be studied outside the discipline of psychology in the future.

The present study extended the previous literature on the cultural value of EM to a more general social context. Encouraged by ancient Chinese philosophies of Confucius’ Analects and I-Ching, EM is a way of self-exertion doctrine that is not limited to the academic domain. Meanwhile, the functions of EM value in different social domains may differ. According to Hwang (1998, 2012) and Fwu et al. (2014), academic EM is connected to filial piety to parents and is an *unconditional positive duty* in Confucian culture, and not EM (TP) is morally wrong. However, in the present study, the data patterns of EM and TP differed in simple main effects of ANOVA, that under an unfair situation, EM was approved, but TP got a neutral score (Table 1). According to these results, in the public’s view, EM in an unfair workplace is not *unconditional*, but *conditional positive duty*, that EM is encouraged but TP is acceptable but not a sin.

In the view of social cognition, there are two motives underlying observers’ evaluating process of EM.First, one’s evaluation is motivated by the cultural value of self-exertion that everyone should make an effort. Second, one could also be motivated by the value of fairness to express their discontent with unfair circumstances, which would help to regard TP as negatively resistant behavior accompanied by frustration or anger emotions toward inequality. The neutral score of TP may result from two opposing motives, which could be measured and tested in further research. Besides, although previous theoretical literature argued that academic EM is an unconditional duty (Hwang, 2012), there may still exist some conditions in people’s moral evaluation process of academic EM, just as the working EM in our research. The moral philosophy of cultural ideals and people’s actual moral cognition process should be differentiated, which could be discovered in further research.

On the results of measurements, the OBE and IBE scales were constructed in the learning virtue research (Chen et al., 2016), and the modified version showed its explanatory power in non-academic topics. Both OBE and IBE correlated positively with EM while negatively with TP, indicating the general influence of cultural value on moral evaluation. As the exception, the correlations were insignificant for effort-making in high return-expectation situations. It could be due to the low *SD*s of Q1 and Q2 (0.59 and 0.76) with a small sample size (*n* = 55) in this situation, which should be clarified with a larger sample size in the future.

A the same time, the two types of effort beliefs did not show a difference in correlating with evaluations of TP/EM behaviors. According to the *Beliefs about Effort* theory (Chen et al., 2016), OBE emphasizes the purpose value of EM itself, while IBE emphasizes the instrumental value of achievements. Thus, the correlations of OBE-EM/TP should be more stable regardless of return expectation, while IBE-EM/TP should be more dependent on the fairness of circumstance, which could not be found in this study. Several possibilities may exist: (1) the OBE and IBE had a 0.63 correlation with each other, and the participants with high IBE scores would have higher OBE scores; (2) the manipulation of return expectation in quasi-experiments was about the short-term return, while the construct of IBE includes both short-term achievement and long-term self-improvement after persistent effort making behaviors. The differences between correlations were not tested directly by moderation in regression analysis (such as Hayes Test) or other similar statistics due to the between-participants design and relatively small sample size. In further research, within-participants design, scenarios with short-term vs. long-term return expectation manipulation and large sample size should be employed to clarify the functional similarities and differences between OBE and IBE in the workplace.

This study also has several limitations. First, the scenarios were designed from a third-party stand, aiming at the public’s evaluation of other people’s TP and EM behaviors, not individuals’ own behavior tendencies. Even the Q2 questions asked the participants to imagine “if you are Zhang,” the answers still could not represent individuals’ own TP or EM behavior tendency in the actual organizational situation, which should be studied in future field research.

Second, there was also literature about hard-working virtue and its functions in western culture in organizational behavior discipline, such as Protestant Work Ethic (Mirels and Garrett, 1971). Meanwhile, there are also similar social phenomena to TP in other societies with different wording, such as Satori Generation in Japan and Sampo Generation in Korea (Arifahsasti and Iskandar, 2022). This article started with a view of Chinese cultural research on China’s social phenomena. At the same time, the interaction effects between hard-working value and return expectation may also be applied to other working ethics and other societies. Further research could explore the value of EM in other East-Asia societies and other hard-working ethics in non-Confucian societies.

Third, our previous qualitative research indicated that Chinese people’s buzz-wording of TP is discussed in both organizational and social contexts (refer to Supplementary material); the latter indicates the high pressure and over-competition with a low return rate in modern China society. In the current quasi-experiment, we only discussed TP/EM behaviors in the workplace. Further research should also investigate the functions of the cultural value of EM in a general social context, such as the issues of apartment-purchase and child-parenting.

Fourth, the sample was collected through an online questionnaire with a relatively high invalid respond rate (26.58%) and potential accessibility bias (Wright, 2005; Van Selm and Jankowski, 2006); thus, the representativeness and generalizability of the results should be verified in an off-line sample of non-internet users.

## Data availability statement

The original data included in this study are available by contact with the corresponding author: xuhanyu@ecupl.edu.cn.

## Ethics statement

Ethical review and approval was not required for the study on human participants in accordance with the local legislation and institutional requirements. Written informed consent from the patients/participants or patients/participants legal guardian/next of kin was not required to participate in this study in accordance with the national legislation and the institutional requirements.

## Author contributions

The author confirms being the sole contributor of this work, and has approved it for publication.
