# Supplementary material for: How do Chinese people evaluate “Tang-Ping” (lying flat) and effort-making: The moderation effect of return expectation
Source: Front Psychol. 2022 Nov 16;13:871439. doi: 10.3389/fpsyg.2022.871439 (PMC9709313; doi:10.3389/fpsyg.2022.871439)
Supplement: Supplementary file 1 [file Data_Sheet_1.docx]

Supplementary Material

# Supplementary Tables

**Supplementary Table 1.** The Chinese version of scenarios.

|  | Low return expectation | High return expectation |
| --- | --- | --- |
| EM | 张三刚从大学毕业，进入了一家互联网公司工作。公司设立了年终绩效奖励，用来激励新进员工的努力奋斗；但张三听说，几乎没有同事获得相应的奖励。  进入公司之后，张三除了完成部门内基本工作量之外，还主动加入了公司的项目组工作、主动加班，在公司绩效排名上名列前茅。 | 张三刚从大学毕业，进入了一家互联网公司工作。公司设立了优渥的年终绩效奖励，用来激励新进员工的努力奋斗；张三听说，曾有不少同事获得了不错的奖励。  进入公司之后，张三除了完成部门内基本工作量之外，还主动加入了公司的项目组工作、主动加班，在公司绩效排名上名列前茅。 |
| TP | 张三刚从大学毕业，进入了一家互联网公司工作。公司设立了年终绩效奖励，用来激励新进员工的努力奋斗；但张三听说，几乎没有同事获得相应的奖励。  进入公司之后，张三除了完成部门内基本工作量之外，基本不参与公司的项目组工作、非必要不加班，在公司绩效排名上成绩平平。 | 张三刚从大学毕业，进入了一家互联网公司工作。公司设立了优渥的年终绩效奖励，用来激励新进员工的努力奋斗；张三听说，曾有不少同事获得了不错的奖励。  进入公司之后，张三除了完成部门内基本工作量之外，基本不参与公司的项目组工作、非必要不加班，在公司绩效排名上成绩平平。 |

**Supplementary Table 2.** The revised OBE and IBE scales.

|  | Chinese item | English Translation | Corresponding item in Wang & Chen (2020, pp. 82-83) |
| --- | --- | --- | --- |
| OBE | 努力**工作**是**每个人**的本分。 | Hard-working is everyone’s duty. | 努力**用功读书**是**学生**的本分。 |
|  | **每个人**都有**努力工作**的责任。 | It is everyone’s responsibility to work hard. | **学生**的责任就是**用功读书**。 |
|  | **人**要为自己不**努力**、偷懒而感到惭愧。 | Someone should feel shame when he/she is lazy and does not work hard. | **学生**要为自己不**用功读书**、偷懒而感到惭愧。 |
|  | **每个人**都该做的事就是努力工作。 | What everyone should do is hard-working. | **学生**该做的事情就是努力**学习**。 |
| IBE | 任何人只要认真拼搏，都可以克服**工作**上的困难。 | Everyone could overcome his/her difficulties in work if working hard. | 任何人只要认真打拼，都可以克服**学习**上的困难。 |
|  | 努力可以突破一个人能力的限制。* | Effort can conquer the limitations of one’s ability. | 努力可以突破一个人能力的限制。 |
|  | 每个人的能力都可以无限地进步。* | One can improve his/her ability with no limitations. | 每个人的能力都可以无限地进步。 |
|  | 只要再接再厉，每个人的能力都不可限量。* | If one makes persistent efforts, his/her ability is unlimited. | 只要再接再厉，每个人的能力都不可限量。 |

Note: Bold characters indicate the revised part. The items with * were not revised for not academic specified. OBE: Obligation-oriented Belief of Effort. IBE: Improvement-oriented Belief of Effort. The original full OBE/IBE scales in Chinese could be found: Wang, K.-H., & Chen, S.-W. (2020). The Predictive Effects of Parents’ Beliefs about Effort on Their Children’s Learning Engagement: Two Mediated Models. *Indigenous Psychological Research in Chinese Societies, 54*, 63-111. doi:10.6254/IPRCS.202012_(54).0002

# Supplementary Figures


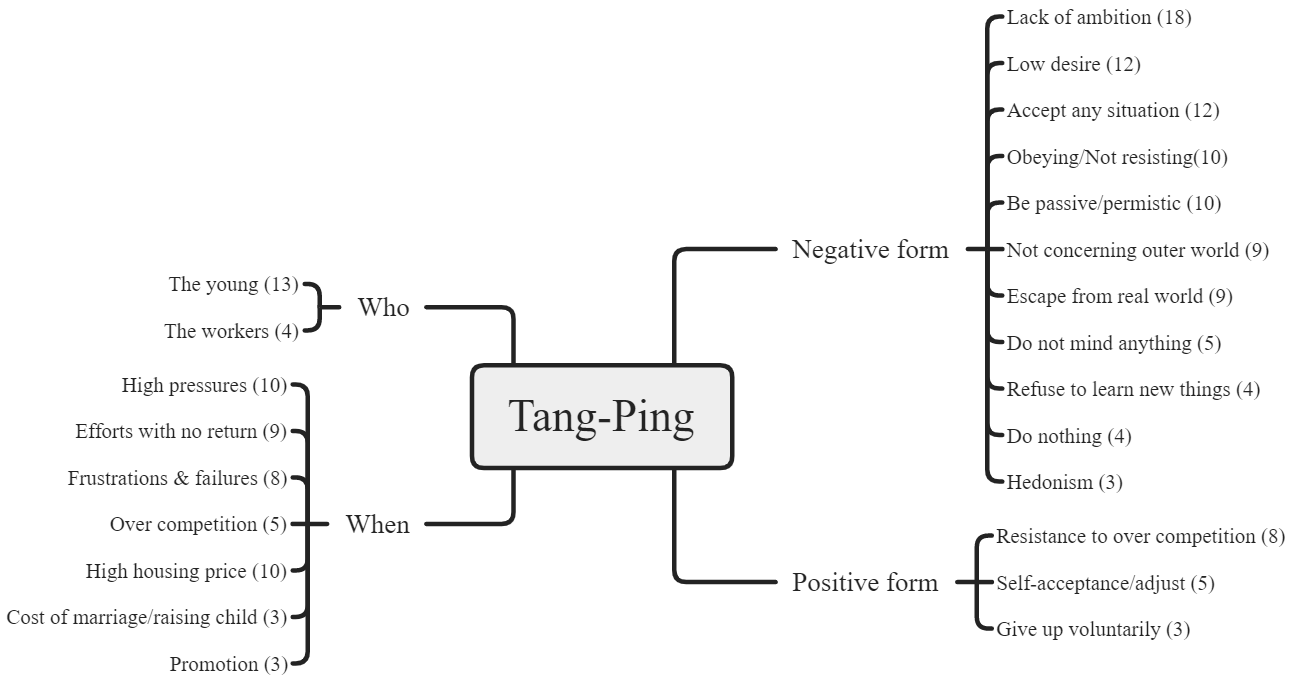


**Supplementary Figure 1.** The phenomenography of TP. The original Chinese version is in the author’s previous manuscript: Hsu, H. Y. (2022). *How do the Public Evaluate "Tang-Ping": The Effects of Cultural Values* [unpublished manuscript]. School of Social Development, East China University of Political Science and Law.
